# Association of sociodemographic, proximal, and distal clinical factors with current suicidal ideation: Findings from a nonclinical sample of young adults

**DOI:** 10.1192/j.eurpsy.2023.14

**Published:** 2023-02-27

**Authors:** Błażej Misiak, Jerzy Samochowiec, Łukasz Gawęda, Dorota Frydecka

**Affiliations:** 1 Department of Psychiatry, Wroclaw Medical University, Wroclaw, Poland; 2 Department of Psychiatry, Pomeranian Medical University, Szczecin, Poland; 3 Experimental Psychopathology Lab, Institute of Psychology, Polish Academy of Sciences, Warsaw, Poland

**Keywords:** childhood trauma, dyslexia, psychotic-like experiences, self-harm, suicide

## Abstract

**Background:**

Accumulating evidence indicates that a variety of distal and proximal factors might impact a risk of suicide. However, the association between both groups of factors remains unknown. Therefore, in the present study, we aimed to investigate the interplay between distal and proximal correlates of the current suicidal ideation.

**Methods:**

A total of 3,000 individuals (aged 18–35 years, 41.7% males), who had reported a negative history of psychiatric treatment, were enrolled through an online computer-assisted web interview. Self-reports were administered to measure: (a) distal factors: a history of childhood trauma (CT), reading disabilities (RDs), symptoms of attention-deficit/hyperactivity disorder (ADHD), lifetime history of non-suicidal self-injury (NSSI), lifetime problematic substance use as well as family history of schizophrenia and mood disorders; (b) proximal factors: depressive symptoms, psychotic-like experiences (PLEs), and insomnia; and (c) sociodemographic characteristics.

**Results:**

Suicidal ideation was directly associated with unemployment, being single, higher level of RD, lifetime history of NSSI as well as higher severity of PLEs, depression, and insomnia. The association of distal factors with suicidal ideation was fully (a history of CT and symptoms of ADHD) or partially (a history of NSSI and RD) mediated by proximal factors (PLEs, depression, and insomnia).

**Conclusions:**

Main findings from this study posit the role of distal factors related to neurodevelopmental disorders, CT and NSSI in shaping suicide risk. Their effects might be partially or fully mediated by depression, PLEs, and insomnia.

## Introduction

Suicide is increasingly being perceived as a complex phenomenon that appears to be the consequence of interactions between multiple psychological, biological, and social factors. It has been shown that people tend to underreport suicidal ideation [1], and most of the suicide attempters deny experiencing suicidal ideation when asked by healthcare providers [2]. Moreover, it has been estimated that more than 50% of suicide decedents saw a health provider within a month before committing suicide [3, 4]. These observations indicate the necessity to improve our understanding of processes leading to suicide in order to develop effective prevention strategies.

To date, a number of theoretical models of suicide have been proposed (for review, see [5]). These include the diathesis–stress model [6, 7], the biopsychosocial model [8], the integrated motivational–volitional model [9], the interpersonal theory of suicide [10], and the narrative crisis model [11]. Some authors argue that suicide risk models inform about mid-term or long-term risk but not about the risk in a short-term perspective, for example, within days or hours [5, 12]. Consequently, there are attempts that aim to shift from simple risk stratification to dynamic planning of interventions. For instance, it has recently been proposed to reformulate suicide risk stratification through the assessment of risk status (individual risk relative to specific subpopulation), risk state (individual risk with respect to baseline assessment or other time points), available resources (internal and social strengths that can support the individual in crisis), and foreseeable changes (circumstances that can quickly increase the risk of suicide) [13]. Nevertheless, this approach still requires the recognition of risk factors in order to offer specific interventions.

There are certain suicide risk models, for example, the narrative crisis model [11] and the biopsychosocial model [8], that posit the necessity to dissect distal risk factors (also known as long-term factors) and proximal risk factors (also known as acute risk factors) in order to better understand the development of suicidal ideation. Distal risk factors are pre-existing vulnerabilities that may facilitate suicidal ideation or behavior [14]. Most frequently, previous studies have shown that the following distal risk factors are associated with suicidal ideation: past suicidal attempts [15], a history of mental illness [16], especially schizophrenia spectrum psychosis [17] and recurrent depressive episodes related to major depressive and bipolar disorder [18], a history of childhood trauma (CT) [19] with sexual abuse playing a significant role [20, 21], a history of non-suicidal self-injury (NSSI) [22, 23], parental death by suicide, parental mental illness, and parental antisocial personality disorder [24]. On the other side, there are several proximal risk factors indicative of an imminent suicide risk identified, including substance use [25], negative interpersonal events [26], negative affective states, such as intense anxiety or agitation [27], insomnia [28], current mental disorders [29] as well as physical suffering [30]. Among them, recent studies highlight the role of psychotic-like experiences (PLEs) that are characterized by low severity, persistence, or associated functional impairment, and thus cannot be the basis to diagnose mental disorders according to international diagnostic systems [31–33]. Primarily, PLEs have been considered to serve as a risk factor of psychotic disorders; however, there is accumulating evidence that their occurrence might also predict the development of other mental disorders [34]. Nevertheless, several studies have shown that PLEs are associated with increased suicide risk [32, 33, 35]. Although distal risk factors may indicate individuals who are more likely to have suicidal thoughts and engage in suicidal behavior in their lifetime, proximal risk factors are indicative of individuals with increased intensity of short-term suicidal ideation and a higher likelihood to act on their suicidal thoughts [26].

There are studies showing that assessment of distal and proximal risk factors might serve as a valid empirical model of progression from chronic to near-term suicidal risk. These studies clearly indicate that pre-existing neurodevelopmental vulnerabilities, including, for example, CT history, trait impulsivity, and perfectionism might interact with proximal factors, for example, suicidal narratives, fear of humiliation, recent life stressors in shaping near-term suicidal risk [36, 37]. However, there is accumulating evidence that other neurodevelopmental vulnerabilities, for example, attention-deficit/hyperactivity disorder (ADHD) symptoms and specific learning disabilities may also serve as prerequisites of trajectories leading to suicide. Indeed, recent meta-analyses found that a diagnosis of ADHD is associated with increased risk of suicidal ideation, plans and attempts as well as completed suicide [38, 39]. Also, it has been found that specific learning disorders, including reading disabilities (RDs), are associated with higher risk of suicidal attempts after adjustment for known risk factors, for example, CT, a history of mental disorders and substance abuse [40]. Nevertheless, specific mechanisms linking these neurodevelopmental vulnerabilities with suicide risk remain unknown. Moreover, their assessment in suicide risk models addressing interactions between proximal and distal risk factors has not been performed so far. Taking into consideration these research gaps in the field, we aimed to investigate the association of the interplay between distal (symptoms of ADHD, RD, CT history, and lifetime history of NSSI) and proximal factors (depressive symptoms, PLEs, and insomnia) with the current suicidal ideation in a nonclinical population.

## Methods

### Participants

The snowball sampling method was used to recruit participants. All questionnaires were administered through the computer-assisted web interview. The survey was posted in social media and survey websites between April and October, 2022. Participants were informed about confidentiality and anonymous character of the survey. All of them represented inhabitants of three large Polish cities, including Szczecin, Warsaw, and Wroclaw. They were included in case of meeting two inclusion criteria, that is, age between 18 and 35 years and a negative lifetime history of psychiatric treatment. The results of the present study are part of a bigger project addressing epigenetic mechanisms of psychosis proneness. The protocol of the study was approved by the Ethics Committees at the Institute of Psychology (Polish Academy of Sciences in Warsaw, Poland, approval number: 16/VII/2022), Wroclaw Medical University (Wroclaw, Poland, approval number: 129/2022), and Pomeranian Medical University (Szczecin, Poland, approval number: KB-006/25/2022).

### Assessments

#### Childhood trauma

A history of four categories of CT (under the age of 17 years), including emotional abuse and neglect, physical abuse as well as sexual abuse was recorded. To obtain information about emotional neglect and abuse, as well as bullying we used three items of the Traumatic Experience Checklist (TEC) [41]. Additionally, three items were selected from the Childhood Experience of Care and Abuse Questionnaire (CECA.Q) to record exposure to sexual abuse [42]. A detailed description of all items is reported in Supplementary Material.

#### Assessment of RD

The Adult Reading Questionnaire (ARQ) was used to assess the severity of RD [43]. The ARQ is a 15-item self-report that includes seven items investigating literacy skills, word-finding abilities, and organization, two items measuring self-appraisal of general reading abilities, two items measuring the frequency or reading and writing, and four items about a diagnosis of dyslexia. The total ARQ score ranges between 0 and 43. Higher ARQ scores indicate worse reading abilities. The Cronbach’s alpha of the ARQ was 0.660 in our sample.

#### Screening of ADHD

The Adult ADHD Self-Report Scale (ASRS) was used to assess ADHD symptoms. It is a self-report questionnaire with nine items measuring inattention and nine items measuring impulsivity/hyperactivity over the period of the preceding 6 months [44]. Each item refers to the frequency of specific symptoms on a five-point scale (0—“never” to 4—“very often”). The total score ranges between 0 and 64. The Cronbach’s alpha of the ASRS was 0.819 in our sample.

#### PLEs

For the purpose of this study, we developed a 16-item questionnaire that records the presence of PLEs during the preceding month. Participants were asked to assess the presence of specific PLEs that cannot be attributed to substance use on a four-point scale (1—“never”; 2—“sometimes”; 3—“often”; and 4—“almost always”). Specific items were obtained from the following questionnaires (for the content of specific items, see Supplementary Material): (a) the Revised Hallucination Scale [45–47] (three items); (b) the Revised Green et al., Paranoid Thoughts Scale [48] (four items); and (c) the Prodromal Questionnaire [49] (nine items). The total score of the questionnaire ranges between 16 and 64 points. The Cronbach’s alpha of the questionnaire was 0.784 in our sample.

#### Depressive symptoms, suicidal ideation, and NSSI

The Patient Health Questionnaire-9 (PHQ-9) was administered to measure the level of depressive symptoms [50]. It includes nine questions about the frequency of specific depressive symptoms over the preceding 2 weeks that are scored on a four-point scale (from 0—“not at all” to 3—“nearly every day”). The Cronbach’s alpha of the PHQ-9 was 0.763 in our sample. The last item: “thoughts that you would be better off dead, or of hurting yourself in some way?” was used to analyze the current suicidal ideation. Participants who scored at least 1 point on this item were classified as those experiencing current suicidal ideation. In turn, a lifetime history of non-suicidal ideation was assessed using one item from the Deliberate Self-Harm Inventory (DSHI) [51]: “Have you ever intentionally (i.e., on purpose) cut your wrist, arms, or other area(s) of your body (without intending to kill yourself)?”

#### Insomnia

The level of insomnia was assessed using four items from the Insomnia Severity Index (ISI) [52]. The ISI is based on seven questions. We used the first three items to record difficulty in falling asleep, difficulty staying asleep, and problems with waking up too early on a five-point scale (scored from 0—“none” to 4—“very severe”). The next question assessed the level of satisfaction/dissatisfaction with the current sleep pattern on a five-point scale (scored from 0—“very satisfied” to 4—“very dissatisfied”). The total score from the ISI questions used in the present study ranged between 0 and 16, with higher scores indicating greater severity of insomnia. The Cronbach’s alpha of this questionnaire was 0.705.

### Data analysis

There were no missing data in the present analysis. Before performing data analysis, variables potentially associated with a risk of suicidal ideation were divided into the following groups: (a) sociodemographic characteristics (age, gender, education, being married or informal relationship, and occupation); (b) distal factors (symptoms of ADHD and RD, lifetime history of NSSI and problematic substance use, family history of schizophrenia and mood disorders as well as CT history); and (c) proximal factors (depressive symptoms, insomnia, and PLEs). Symptoms of ADHD and RD were included as distal factors as they represent disorders with the onset in the early neurodevelopmental period.

**Figure 1.**
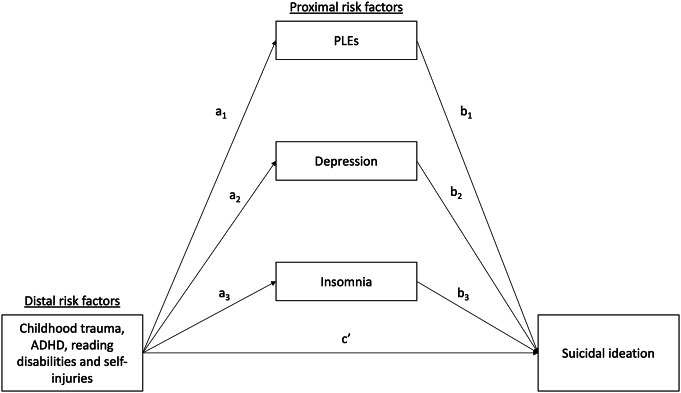
The mediation model tested in the present study. Results of data analysis are reported in Table 3.

A series of bivariate comparisons of individuals with and without the current suicidal ideation were performed using the χ^2^ (categorical variables) or the Mann–Whitney *U* test (continuous variables). Next, binary logistic regression analyses were performed in a step-by-step procedure. The current suicidal ideation was included as a dependent variable. First, we tested the effects of proximal factors, distal factors, and sociodemographic characteristics as independent variables separately. Then, we combined these sets of variables in two blocks, that is, distal and proximal factors with and without sociodemographic characteristics. The Hosmer–Lemeshow test was implemented to assess the goodness of fit for logistic regression models. In this part, the results of data analysis were considered statistically significant in case of a *p*-value lower than 0.05. Finally, we analyzed the mediating effect of proximal factors in the association between distal factors and suicidal ideation using the PROCESS Macro (model 4) [53]. Mediation analyses were performed separately for specific distal factors (Figure 1). The results of this analysis were considered significant if 95% CI did not include zero.

## Results

### The general characteristics of the sample

A total of 3,000 young adults were enrolled (aged 25.4 ± 4.9 years, 41.7% males). General characteristics of the sample are shown in Table 1. Individuals reporting suicidal ideation were significantly younger, were more likely to be females, had lower education level, reported worse occupational situation as well as were less likely to be married or in an informal relationship compared to their counterparts. Family history of schizophrenia and mood disorders, lifetime problematic substance use, exposure to any CT, as well as lifetime history of NSSI were significantly more frequent among individuals reporting suicidal ideation. Moreover, participants reporting suicidal ideation had higher levels of RD, ADHD symptoms, depression, insomnia, and PLEs.

**Table 1.** Descriptive characteristics of the sample.

	Total sample	SI(+), *n* = 828	SI(−), *n* = 2,172	*p*
Age	25.4 ± 4.9	25.1 ± 4.9	25.5 ± 4.9	0.035
Gender, males	1,250 (41.7)	301 (36.4)	949 (43.7)	<0.001
Education				
Primary	101 (3.4)	48 (5.8)	53 (2.4)	<0.001
Vocational	46 (1.5)	22 (2.7)	24 (1.1)	
Secondary	911 (30.4)	281 (33.9)	630 (29.0)	
Incomplete higher	720 (24.0)	189 (22.8)	531 (24.5)	
Higher	1,222 (40.7)	288 (34.8)	934 (43.0)	
Occupation				
Unemployed	113 (3.8)	44 (5.3)	69 (3.2)	<0.001
Student	1,557 (51.9)	468 (56.5)	1,089 (50.1)	
Employed	1,300 (43.3)	303 (36.6)	997 (45.9)	
Rent	30 (1.0)	13 (1.6)	17 (0.8)	
Married or informal relationship	1,999 (66.6)	487 (58.8)	1,512 (69.6)	<0.001
Family history of schizophrenia	146 (4.9)	56 (6.8)	90 (4.1)	0.002
Family history of mood disorder	597 (19.9)	202 (24.4)	395 (18.2)	<0.001
Lifetime problematic substance use	1,115 (37.2)	354 (42.8)	761 (35.0)	<0.001
CT	2,680 (89.3)	779 (94.1)	1,901 (87.5)	<0.001
ARQ	13.2 ± 5.7	15.7 ± 5.9	12.2 ± 5.4	<0.001
Self-injuries, lifetime	651 (21.7)	308 (37.2)	343 (15.8)	<0.001
ASRS	30.2 ± 10.3	34.4 ± 10.3	28.6 ± 10.8	<0.001
PLEs	22.5 ± 4.9	25.0 ± 6.0	21.5 ± 4.0	<0.001
PHQ-9	9.0 ± 4.7	12.3 ± 4.7	7.7 ± 4.1	<0.001
ISI	5.8 ± 3.7	7.7 ± 3.8	5.1 ± 3.4	<0.001

Data presented as mean ± SD or *n* (%).

Abbreviations: ARQ, the Adult Reading Questionnaire; ASRS, the Adult ADHD Self-Report Scale; CT, childhood trauma; ISI, the Insomnia Severity Index; PHQ-9; the Patient Health Questionnaire-9; PLEs, psychotic-like experiences; SI(+), participants with the current suicidal ideation; SI(−), participants without the current suicidal ideation.

### Results of binary logistic regression analyses

The results of the binary logistic regression analyses are presented in Table 2. The following factors were significantly associated with a higher risk of suicidal ideation in separate blocks of independent variables: (a) sociodemographic characteristics: female gender, being single, lower level of education and being unemployed, or rent; (b) all proximal factors, that is, higher levels of PLEs, depression, and insomnia; and (c) distal factors: exposure to any CT, lifetime history of NSSI as well as higher levels of ADHD symptoms and RD. After combining all factors together, the following of them were associated with a higher risk of suicidal ideation: (a) sociodemographic characteristics: being single and being unemployed or rent; (b) all proximal factors, that is, higher levels of PLEs, depression, and insomnia; and (c) distal factors: higher levels of RD and lifetime history of NSSI.

**Table 2.** Results of binary logistic regression analysis.

Model	Risk factor	OR	95% CI	*p*
Sociodemographic characteristics (Nagelkerke *R*^2^ = 0.039)	Age	0.991	0.975–1.008	0.322
	Gender, male	0.710	0.600–0.839	<0.001
	Education, higher or incomplete higher	0.685	0.578–0.812	<0.001
	Occupational situation, employed or student	0.536	0.377–0.763	<0.001
	Married or informal relationship	0.612	0.516–0.725	<0.001
Distal risk factors (Nagelkerke *R*^2^ = 0.178)	CT	1.507	1.077–2.109	0.017
	ASRS	1.035	1.025–1.045	<0.001
	ARQ	1.078	1.061–1.096	<0.001
	NSSI, lifetime	2.470	2.032–3.002	<0.001
	Problematic substance use, lifetime	1.060	0.887–1.267	0.520
	Family history of schizophrenia	1.207	0.825–1.765	0.333
	Family history of mood disorder	1.080	0.872–1.337	0.480
Proximal risk factors (Nagelkerke *R*^2^ = 0.302)	PLEs	1.071	1.048–1.093	<0.001
	PHQ-9	1.201	1.173–1.229	<0.001
	ISI	1.096	1.068–1.126	<0.001
Distal and proximal risk factors (Nagelkerke *R*^2^ = 0.331)	CT	1.127	0.790–1.607	0.509
	ASRS	0.993	0.982–1.005	0.251
	ARQ	1.055	1.036–1.074	<0.001
	NSSI, lifetime	1.930	1.557–2.392	<0.001
	Problematic substance use, lifetime	0.999	0.824–1.212	0.993
	Family history of schizophrenia	1.245	0.817–1.897	0.307
	Family history of mood disorder	0.864	0.682–1.095	0.226
	PLEs	1.053	1.030–1.077	<0.001
	PHQ-9	1.182	1.152–1.212	<0.001
	ISI	1.098	1.069–1.128	<0.001
Proximal and distal risk factors; sociodemographic characteristics (Nagelkerke *R*^2^ = 0.341)	CT	1.119	0.784–1.597	0.537
	ASRS	0.996	0.985–1.008	0.518
	ARQ	1.050	1.031–1.070	<0.001
	NSSI, lifetime	1.955	1.567–2.439	<0.001
	Problematic substance use, lifetime	1.030	0.848–1.252	0.766
	Family history of schizophrenia	1.161	0.762–1.770	0.488
	Family history of mood disorder	0.849	0.668–1.079	0.180
	PLEs	1.053	1.029–1.077	<0.001
	PHQ-9	1.175	1.146–1.206	<0.001
	ISI	1.097	1.068–1.128	<0.001
	Age	0.997	0.978–1.017	0.771
	Gender, male	0.954	0.783–1.161	0.637
	Education, higher or incomplete higher	0.860	0.705–1.051	0.140
	Occupation, employed or student	0.579	0.385–0.870	0.009
	Married or informal relationship	0.652	0.535–0.793	<0.001

Abbreviations: ARQ, the Adult Reading Questionnaire; ASRS, the Adult ADHD Self-Report Scale; CT, childhood trauma; ISI, the Insomnia Severity Index; NSSI, non-suicidal self-injuries; PHQ-9; the Patient Health Questionnaire-9; PLEs, psychotic-like experiences.

### Results of mediation analyses

The results of mediation analyses are shown in Table 3. In these analyses, we further tested the effect of distal mediators that appeared to be significantly associated with a risk of suicidal ideation in any logistic regression analysis. All direct effects of distal factors on proximal factors were significant. Also, all direct effects of proximal factors on the risk of suicidal ideation were significant. However, the direct effects of distal factors on the risk of suicidal ideation were significant only in the case of a lifetime history of NSSI and RD. In other words, the direct effects of CT history and ADHD symptoms on the risk of suicidal ideation were not significant. All indirect effects, that is, from distal factors to the risk of suicidal ideation through the effects of proximal factors, were significant. The mediation models explained 30.9–32.2% of the variance in the risk of suicidal ideation.

**Table 3.** Results of mediation analysis.

Distal risk factor	Path	*B*	SE	95% CI
CT (*R*^2^ = 0.309)	Direct effect of CT on PLEs (a_1_)	2.229	0.282	1.675–2.782
	Direct effect of CT on depression (a_2_)	2.496	0.275	1.957–3.036
	Direct effect of CT on insomnia (a_3_)	1.677	0.216	1.253–2.100
	Direct effect of PLEs on suicidal ideation (b_1_)	0.063	0.011	0.041–0.084
	Direct effect of depression on suicidal ideation (b_2_)	0.182	0.012	0.158–0.206
	Direct effect of insomnia on suicidal ideation (b_3_)	0.090	0.014	0.063–0.116
	Direct effect of CT on suicidal ideation (c’)	0.213	0.178	−0.137–0.563
	Indirect effect of CT (through PLEs) on suicidal ideation (a_1_b_1_)	0.140	0.030	0.084–0.201
	Indirect effect of CT (through depression) on suicidal ideation (a_2_b_2_)	0.454	0.058	0.349–0.574
	Indirect effect of CT (through insomnia) on suicidal ideation (a_3_b_3_)	0.150	0.029	0.099–0.213
ADHD (*R*^2^ = 0.309)	Direct effect of ADHD on PLEs (a_1_)	0.206	0.008	0.191–0.221
	Direct effect of ADHD on depression (a_2_)	0.234	0.007	0.220–0.248
	Direct effect of ADHD on insomnia (a_3_)	0.106	0.006	0.094–0.119
	Direct effect of PLEs on suicidal ideation (b_1_)	0.061	0.011	0.039–0.083
	Direct effect of depression on suicidal ideation (b_2_)	0.180	0.013	0.155–0.204
	Direct effect of insomnia on suicidal ideation (b_3_)	0.090	0.014	0.064–0.117
	Direct effect of ADHD on suicidal ideation (c’)	0.004	0.006	−0.007–0.015
	Indirect effect of ADHD (through PLEs) on suicidal ideation (a_1_b_1_)	0.013	0.003	0.008–0.018
	Indirect effect of ADHD (through depression) on suicidal ideation (a_2_b_2_)	0.042	0.003	0.036–0.049
	Indirect effect of ADHD (through insomnia) on suicidal ideation (a_3_b_3_)	0.010	0.002	0.007–0.013
RD (*R*^2^ = 0.320)	Direct effect of RD on PLEs (a_1_)	0.283	0.015	0.255–0.312
	Direct effect of RD on depression (a_2_)	0.286	0.014	0.258–0.314
	Direct effect of RD on insomnia (a_3_)	0.148	0.012	0.125–0.170
	Direct effect of PLEs on suicidal ideation (b_1_)	0.052	0.011	0.030–0.073
	Direct effect of depression on suicidal ideation (b_2_)	0.172	0.012	0.148–0.196
	Direct effect of insomnia on suicidal ideation (b_3_)	0.090	0.014	0.063–0.117
	Direct effect of RD on suicidal ideation (c’)	0.049	0.009	0.032–0.067
	Indirect effect of RD (through PLEs) on suicidal ideation (a_1_b_1_)	0.015	0.003	0.008–0.022
	Indirect effect of RD (through depression) on suicidal ideation (a_2_b_2_)	0.049	0.004	0.041–0.058
	Indirect effect of RD (through insomnia) on suicidal ideation (a_3_b_3_)	0.013	0.002	0.009–0.018
NSSI (*R*^2^ = 0.322)	Direct effect of NSSI on PLEs (a_1_)	2.313	0.215	1.892–2.734
	Direct effect of NSSI on depression (a_2_)	2.829	0.208	2.422–3.236
	Direct effect of NSSI on insomnia (a_3_)	1.027	0.166	0.701–1.353
	Direct effect of PLEs on suicidal ideation (b_1_)	0.058	0.011	0.037–0.080
	Direct effect of depression on suicidal ideation (b_2_)	0.173	0.012	0.149–0.197
	Direct effect of insomnia on suicidal ideation (b_3_)	0.093	0.014	0.066–0.120
	Direct effect of NSSI on suicidal ideation (c’)	0.228	0.032	0.164–0.291
	Indirect effect of NSSI (through PLEs) on suicidal ideation (a_1_b_1_)	0.135	0.031	0.079–0.201
	Indirect effect of NSSI (through depression) on suicidal ideation (a_2_b_2_)	0.489	0.052	0.397–0.598
	Indirect effect of NSSI (through insomnia) on suicidal ideation (a_3_b_3_)	0.096	0.022	0.057–0.143

Covariates include age, sex, being married or in a relationship, education level, and the current occupational situation.

Abbreviations: ADHD, attention-deficit/hyperactivity disorder; CT, childhood trauma; NSSI, non-suicidal self-injuries; RDs, reading disabilities.

## Discussion

In the present study, we found that both sociodemographic factors (being unemployed, single, and male), as well as clinical characteristics (CT history, RD, ADHD, lifetime history of NSSI, PLEs, depression, and insomnia), play a role in the development of suicidal ideation. However, further analysis showed that the effects of CT history and ADHD symptoms on the risk of suicidal ideation are fully mediated by the effects of proximal factors, including PLEs, depression, and insomnia. In turn, other distal factors, including RD and NSSI may directly and indirectly (through proximal factors) increase the risk of suicidal ideation. To the best of our knowledge, this is, the first investigating potential mechanisms by which symptoms of ADHD and RD together with other distal factors interact with proximal factors represented by depressive symptoms, PLEs, and insomnia to shape the current suicidal ideation. Previous studies have investigated only some of variables that we have combined in our model providing grounds for more comprehensive analysis of the interplay and significance of proximal and distal factors.

Childhood traumatic events, especially a history of sexual or physical abuse, have been shown to cause at least a threefold increase in lifetime suicide attempts and lifetime suicidal ideation [54–56]. Some studies suggest that suicidal risk is associated independently with childhood adverse experiences [54, 55, 57]. However, such experiences predispose to various mental disorders, including mood, anxiety, substance use, and borderline personality disorders [58–60], all of which may act as mediators for suicidal behavior [61, 62]. Moreover, childhood adversities are also associated with earlier onset and more chronic course of mental disorders [63, 64]. Thus, controlling for various dimensions of symptomatology and examining causal pathways as well as possible indirect effects between CT and suicidality can yield reliable results. This is in line with other studies showing that psychopathology associated with childhood adversities partially [65] or fully [66, 67] mediates the relationship between exposure to CT and suicidal ideation or attempts.

In our study, we found that CT history is associated with the risk of suicidal thoughts through the mediating effect of the severity of PLEs, depression, and insomnia. This is in agreement with previous studies, showing that PLEs increase the risk of suicide [68–70]. Moreover, in the longitudinal perspective, baseline severity of PLEs has been observed to predict the worsening of suicidal ideation and behavior over time [32]. Also, associations of delusional ideation and perceptual distortions with suicidality have been observed to be stronger with time and more pronounced in comparison to general psychopathology [32]. Similar to our results, it has been shown that PLEs are predictive of suicidal thoughts, even when controlling for sociodemographic variables and traumatic experiences [71]. Additionally, a history of childhood adversities, such as peer bullying, poverty, and left-behind status, has been found to increase suicide risk through the mediating effect of PLEs [72]. In line with our findings, depressive symptomatology has been associated with a history of CT and suicidality [73], and might mediate the relationship between a history of CT and suicidal risk [66, 74]. Early-life stressors have also been demonstrated to alter sleep regulation, leading to life-long sleep disturbances, including insomnia [75]. On the other site, insomnia has been shown to play a role in dysregulating the systems involved in mood and emotion regulation, thus influencing the onset and maintenance of depressive symptomatology [76]. Insomnia has also been demonstrated to play an important role in the relationship between early-life stressors and depression, thereby influencing the risk of suicidal ideation [77].

Our results on the significance of the association between ADHD symptoms and the risk of suicidal ideation are in line with a recent systematic review and meta-analysis [78]. Moreover, we observed that the relationship between ADHD symptoms and suicidality is fully mediated through proximal factors—PLEs, depression, and insomnia. Follow-up studies of ADHD children into adolescence, as well as early adulthood, indicate that the symptoms of ADHD frequently persist and are associated with various types of psychopathology and dysfunction in later life [79]. It has been demonstrated that the majority of individuals with ADHD experience at least one comorbid mental disorder [80]. The results of our study are in agreement with numerous reports showing that the association between ADHD traits and suicidality is fully mediated by the presence of comorbid mental disorders. This has been shown both with respect to the current [81, 82] as well as lifetime suicidal ideation [83]. Some studies suggest that symptoms of ADHD, such as inattention, impulsivity or executive dysfunction, serve as an independent risk factor for suicidality [84–86]. However, these traits have been found to mainly predict suicide attempts and to a much lesser extent, suicidal ideation [86]. Nevertheless, some studies have observed direct associations of ADHD with suicidality after controlling for comorbid psychiatric disorders [87–89]. To the best of our knowledge, none of the previous studies has tested the mediating effect of PLEs in the association between ADHD symptoms and suicidality. However, one study revealed the moderating effect of emotion dysregulation and impulsivity on the relationship between PLEs and suicidal ideation in children [90].

In our study, we found that RD are directly and indirectly associated with the risk of suicidal ideation. Dyslexia is a developmental disability characterized by difficulties in reading comprehension and writing without overall sensory or intelligence deficits [91]. Accumulating evidence shows that individuals with dyslexia are at greater risk of developing numerous psychological [92] and psychiatric [93] comorbidities. They have the tendency to report lower self-esteem and self-concept, aggressiveness and rule-breaking behaviors, problems in social interactions, as well as higher levels of anxiety and depressive symptoms, fatigue, and sleeping difficulties in comparison with their peers without RD [94, 95]. However, there is a scarcity of studies showing the association between RD and suicidality. It has been demonstrated that children with dyslexia report more suicidal ideation compared to children without RD [96], and adolescents with poor reading abilities might be more likely to experience suicidal ideations or attempts, even after controlling for sociodemographic factors and psychiatric comorbidity [97]. Additionally, the analysis of a broader range of learning disabilities, including impairments of various functional and academic skills, has revealed their association with a higher risk of suicidal ideation and behaviors [98, 99].

Another observation from our study is that the lifetime history of NSSI is directly and indirectly associated with the risk of suicidal ideation. Importantly, logistic regression analysis revealed that lifetime history of NSSI was associated with about twofold higher risk of suicidal ideation, and this magnitude of the effect was the highest among all potential correlates analyzed in the present study. By definition, NSSI describes deliberate behaviors that inflict pain and/or cause damage to the body without suicidal intent [100]. It has been shown that NSSI might be characterized by a variety of inter- and intrapersonal purposes, mainly emotion regulation, communicating distress or influencing behavior of other people [101]. There are numerous studies showing the relationship between NSSI and the increased likelihood of suicide ideation and attempts [102–104]. A recent meta-analysis estimated the risk of fatal outcomes of NSSI at 1.3% within 1 year; however, this risk appeared to be almost threefold higher in a 3-year follow-up [105]. Also, it has been found that the increased risk of suicide following NSSI in longitudinal studies is associated with male gender, multiple episodes of NSSI, a diagnosis of psychiatric disorder, and physical illness [106]. It has been suggested that the relationship between NSSI and increased suicidal risk can be explained by the acquired capability for suicide, whereby repetitive exposure to emotionally provocative and physically painful events results in increased tolerance to pain and decreased fear of death [107]. The results of our study are in line with reports showing the association of NSSI with PLEs and depressive symptomatology [108, 109], which in turn are linked to increased risk of suicidal ideations and behaviors [68, 71].

The results of our study should be interpreted bearing in mind several limitations. First, internet-based surveys can be characterized by a variety of limitations related to insufficient accuracy of self-reports, difficulties in tracking non-response rates and a selection bias [110, 111]. Second, the cross-sectional design limits the possibility of concluding about firm causal inferences. Third, recall biases may exist with respect to assessments of childhood maltreatment, especially in the presence of depressive symptoms and PLEs. Fourth, although assessments in our study included an extensive set of potential factors, the percentage of variance in suicidal ideation explained by our models was limited, suggesting the importance of other mechanisms and factors that were not recorded. Fifth, the results of the present study should be interpreted with caution due to the use of a nonclinical sample and selected items from assessment tools. Therefore, generalization of findings over the clinical population of patients with dyslexia or ADHD should be approached cautiously. At this point, it is also important to note that assessment of suicidal ideation was limited to the use of one item from the PHQ-9. However, there are studies showing the usefulness of this item in stratifying the risk of suicide attempt [2, 112, 113]. Finally, our sample was limited to young adults, and thus generalization to other populations should be approached cautiously.

In sum, the findings of this study demonstrate that the risk of suicide ideation is associated with the interplay of distal (CT, ADHD symptoms, RD, and self-injuries) and proximal factors (PLEs, depression, and insomnia). Longitudinal studies in clinical samples are needed to confirm our findings before their translation into clinical practice. Nevertheless, implications from the present study suggest that patients with neurodevelopmental disorders should be carefully assessed for their suicidal risk, and the current psychiatric symptoms should be treated accordingly in order to decrease the risk of suicidal behaviors.

## Data Availability

Data used in the present study are available in the Open Science Framework (OSF) database (https://osf.io/8ju6b/).

## Supplementary material

For supplementary material accompanying this paper visit http://doi.org/10.1192/j.eurpsy.2023.14. click here to view supplementary material
