# Supplementary material for: Association of sociodemographic, proximal, and distal clinical factors with current suicidal ideation: Findings from a nonclinical sample of young adults
Source: Eur Psychiatry. 2023 Feb 27;66(1):e29. doi: 10.1192/j.eurpsy.2023.14 (PMC10044310; doi:10.1192/j.eurpsy.2023.14)
Supplement: Supplementary file 1 [file S0924933823000147sup001.docx]

**Supplementary Material**

**Measures**

*Childhood trauma*

To obtain information about emotional neglect and abuse, we used two items of the Traumatic Experience Checklist (TEC) [1]: 1) “When you were a child or a teenager have you ever felt emotionally neglected (e.g., being left alone, insufficient affection) by your parents, brothers or sisters?” and 2) “When you were a child or a teenager have you ever felt emotionally abused (e.g., being belittled, teased, called names, threatened verbally, or unjustly punished) by your parents, brothers or sisters?”. One item from the TEC was administered to record the exposure to bullying: “When you were a child or teenager, did you experience psychological violence (e.g., nicknames, teasing) or physical abuse (e.g., jerking, beating) from your peers?”. In turn, three items were selected from the Childhood Experience of Care and Abuse Questionnaire (CECA.Q) to record exposure to sexual abuse [2]: 1) “When you were a child or teenager did you have any unwanted sexual experiences?”; 2) “Did anyone force you or persuade you to have sexual intercourse against your wishes before age 17?” and 3) “Can you think of any upsetting sexual experiences before age 17 with a related adult or someone in authority e.g., teacher?”.

*Psychotic-like experiences (PLEs)*

Specific items were obtained from the following questionnaires: 1) the Revised Hallucination Scale [3-5]: “I hear voice speaking my thoughts aloud”, „I hear people call my name and find that nobody has done so” and „I see shadows and shapes when there is nothing there”; 2) the Revised Green et al., Paranoid Thoughts Scale [6]: “I spent time thinking about friends gossiping about me”, “People wanted me to feel threatened, so they stared at me”, “I was convinced there was a conspiracy about me”, “I was distressed by being persecuted” and “People have been dropping hints for me” and 3) the Prodromal Questionnaire [7]: “When I look at a person, or look at myself in a mirror, I have seen the face change right before my eyes”, “I have heard things other people can’t hear like voices of people whispering or talking”, “I often feel that other have it in for me”, “I have seen things that other people apparently can’t see”, “I have had the sense that some person or force is around me, even though I could not see anyone”, “I sometimes see special meanings in advertisements, shop windows, or in the way things are arranged around me”, “I sometimes smell or taste things that other people can’t smell or taste” and “I often seem to live through events exactly as they happened before”. The total score of the questionnaire ranges between 16 and 64 points. The Cronbach’s alpha of the questionnaire was 0.784 in our sample.

*Other measures*

Personal and family history of psychiatric treatment were recorded using the following questions: “Have you ever been treated for psychiatric disorders?”; “Has anybody from your family received the treatment for schizophrenia?” and “Has anybody from your family received the treatment for depression or bipolar disorder?”. In case of positive responses to the latter two questions, the participants were asked to indicate who has received the treatment. Lifetime problematic substance use was assessed using the screening question from the Mini-International Neuropsychiatric Interview (M.I.N.I.) [8].

**References**

[1] Nijenhuis E, Van der Hart O, Kruger K. The psychometric characteristics of the traumatic experiences checklist (TEC): first findings among psychiatric outpatients. Clin Psychol Psychother. 2002;9:2000-10.

[2] Bifulco A, Bernazzani O, Moran PM, Jacobs C. The childhood experience of care and abuse questionnaire (CECA.Q): validation in a community series. Br J Clin Psychol. 2005;44(Pt 4):563-81. <https://doi.org/10.1348/014466505X35344>.

[3] Gaweda L, Kokoszka A. [Polish version of the Revised Hallucination Scale (RHS) by Morrison et al. Its factor analysis and the prevalence of hallucinatory-like experiences among healthy participants]. Psychiatr Pol. 2011;45(4):527-43.

[4] Morrison AP, Wells A, Nothard S. Cognitive factors in predisposition to auditory and visual hallucinations. Br J Clin Psychol. 2000;39(1):67-78. <https://doi.org/10.1348/014466500163112>.

[5] Morrison AP, Wells A, Nothard S. Cognitive and emotional predictors of predisposition to hallucinations in non-patients. Br J Clin Psychol. 2002;41(Pt 3):259-70. <https://doi.org/10.1348/014466502760379127>.

[6] Freeman D, Loe BS, Kingdon D, Startup H, Molodynski A, Rosebrock L, et al. The revised Green et al., Paranoid Thoughts Scale (R-GPTS): psychometric properties, severity ranges, and clinical cut-offs. Psychol Med. 2021;51(2):244-53. <https://doi.org/10.1017/S0033291719003155>.

[7] Ising HK, Veling W, Loewy RL, Rietveld MW, Rietdijk J, Dragt S, et al. The validity of the 16-item version of the Prodromal Questionnaire (PQ-16) to screen for ultra high risk of developing psychosis in the general help-seeking population. Schizophr Bull. 2012;38(6):1288-96. <https://doi.org/10.1093/schbul/sbs068>.

[8] Sheehan DV, Lecrubier Y, Sheehan KH, Amorim P, Janavs J, Weiller E, et al. The Mini-International Neuropsychiatric Interview (M.I.N.I.): the development and validation of a structured diagnostic psychiatric interview for DSM-IV and ICD-10. J Clin Psychiatry. 1998;59 Suppl 20:22-33;quiz 4-57.
